# Paternity sharing in insects with female competition for nuptial gifts

**DOI:** 10.1002/ece3.9463

**Published:** 2022-10-30

**Authors:** Jessica H. Browne, Darryl T. Gwynne

**Affiliations:** ^1^ Department of Ecology and Evolutionary Biology University of Toronto Mississauga Mississauga Ontario Canada; ^2^ Department of Biology Mount Allison University Sackville New Brunswick Canada

**Keywords:** female competition, nuptial gifts, parental investments, paternity, sperm competition

## Abstract

Male parental investment is expected to be associated with high confidence of paternity. Studies of species with exclusive male parental care have provided support for this hypothesis because mating typically co‐occurs with each oviposition, allowing control over paternity and the allocation of care. However, in systems where males invest by feeding mates (typically arthropods), mating (and thus the investment) is separated from egg‐laying, resulting in less control over insemination, as male ejaculates compete with rival sperm stored by females, and a greater risk of investing in unrelated offspring (cuckoldry). As strong selection on males to increase paternity would compromise the fitness of all a female's other mates that make costly nutrient contributions, paternity sharing (males not excluded from siring offspring) is an expected outcome of sperm competition. Using wild‐caught females in an orthopteran and a dipteran species, in which sexually selected, ornamented females compete for male nuptial food gifts needed for successful reproduction, we examined paternity patterns and compared them with findings in other insects. We used microsatellite analysis of offspring (lifetime reproduction in the orthopteran) and stored sperm from wild‐caught females in both study species. As predicted, there was evidence of shared paternity as few males failed to sire offspring. Further support for paternity sharing is the lack of last‐male sperm precedence in our study species. Although paternity was not equal among sires, our estimates of paternity bias were similar to other insects with valuable nuptial gifts and contrasted with the finding that males are frequently excluded from siring offspring in species where males supply little more than sperm. This suggests paternity bias may be reduced in nuptial‐gift systems and may help facilitate the evolution of these paternal investments.

## Graphical abstract

Relative to systems where males invest in offspring via paternal care, paternity assurance appears to be more complicated in systems where males feed their mates. In this manuscript, we test the hypothesis that paternity is shared in systems where males invest in offspring via nuptial gifts. Consistent across two study species where females rely on male‐provided nutrition, we find evidence of reduced paternity failure suggesting that shared paternity may help facilitate the evolution of nuptial gifts.

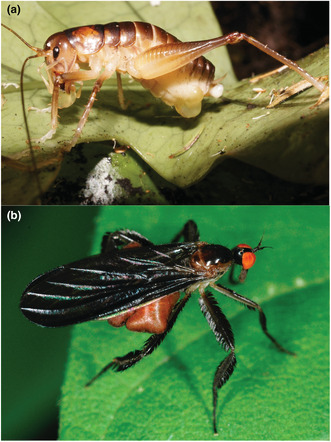

## 1 INTRODUCTION

Although females typically invest more in offspring than males, males of some species can contribute to offspring fitness via paternal care or by feeding their mates (Gwynne, 1991, 2016; Janicke et al., 2016; Trivers, 1972). Paternal care, found in some fish, frogs, and birds, consists of behaviors such as nest building, brooding, feeding, or protecting offspring (Clutton‐Brock, 1991). Nutritional donations provided during courtship or copulation (known as nuptial gifts) are more common in invertebrates and consist of a male's own body parts, secretions, or prey items that benefit a female and her offspring (Thornhill, 1976; reviewed in Vahed, 1998; Lewis et al., 2014). Such investments (paternal effort; Gwynne, 1984a; Thornhill, 1976) can be costly for males and limit their ability to invest in future mates (Gwynne, 1990; Trivers, 1972), yet the direct benefits to females and their offspring can lead to females competing for multiple matings (Bonduriansky, 2001; Gwynne & Simmons, 1990; Herridge et al., 2016). In some species, this competition results in strong sexual selection and the evolution of secondary sexual traits in females (Gwynne, 1981, 1991, 2016; Gwynne & Simmons, 1990; Hare & Simmons, 2018; Herridge et al., 2016; Simmons, 1992; Thornhill, 1979; Tobias et al., 2012; Trivers, 1972).

Central to parental investment theory (Møller & Birkhead, 1993; Requena & Alonzo, 2017; Trivers, 1972; Westneat & Sherman, 1992), males that contribute to offspring are expected to have high confidence of paternity to avoid the fitness costs of cuckoldry (investment in unrelated offspring). Thus, high paternity confidence is thought to facilitate the evolution of paternal effort and investing males will be under strong selection to increase their confidence of paternity (Møller & Birkhead, 1993; Requena & Alonzo, 2017; Trivers, 1972; Westneat & Sherman, 1992). This appears to be the case in many species with paternal care, as insemination is controlled by the male and typically precedes the investment, allowing males to have high confidence in the paternity of the offspring they care for. Examples include pipefish, where unfertilized eggs are placed in an enclosed male brood pouch before undergoing a prolonged pregnancy (Jones & Avise, 2001) or when there are repeated copulations prior to each egg laid, as is the case in *Abetus* water bugs that rear eggs on their backs (Smith, 1979) and sequentially polyandrous birds that help care for young (Delehanty et al., 1998; Møller & Birkhead, 1991; Owens et al., 1995; Schamel et al., 2004).

In systems with nuptial gifts, however, males provide the investment during courtship or copulation (Lewis et al., 2014) and oviposition/fertilization occurs separately, resulting in less direct control over paternity and thus greater potential for cuckoldry. When females mate with multiple males prior to oviposition (common among arthropods; Arnqvist & Nilsson, 2000; Eberhard, 1985; Simmons, 2001), paternity is achieved in competition with rival ejaculates that have been stored within a specialized sperm storage organ (Parker, 1970; Simmons, 2001). Thus, selection for paternity has led to a variety of traits that allow males to bias fertilizations in their favor (Eberhard, 1985; Lloyd, 1979; Parker, 1970; Simmons, 2001). These traits include increased sperm quantity or motility that increases fertilization success (Parker et al., 2017; Rowe & Pruett‐Jones, 2011), attractive male phenotypes that result in preferential sperm usage by the female (Albo et al., 2013; Fedina, 2007; Lüpold et al., 2013; Pizzari & Birkhead, 2000), as well as behaviors that reduce the intensity of sperm competition such as mate guarding, mating plugs, or removal of rival sperm (Birkhead, 1979; Simmons, 2001; Waage, 1979). As a result, paternity is frequently biased in favor of, for example, the highest‐quality males or those that were the last to mate with a female prior to laying eggs (Birkhead & Hunter, 1990; Eberhard, 1985; Lloyd, 1979; Parker, 1970; Simmons, 2001).

Given that investing males are expected to have a high confidence of paternity, one hypothesis is that insect paternity will be highly biased when males invest in nutritious nuptial gifts, possibly via last male sperm precedence due to its prevalence in insects (Gwynne, 1984a; Simmons, 2001). On the other hand, while such biased paternity benefits the successful males, it results in cuckoldry for all other mating males who have also invested substantially in a female's offspring (Good et al., 2006) and may not be able to mate again for several days (Gwynne, 1990; Perry & Tse, 2013). Thus, an alternative hypothesis to that of highly biased paternity is that sperm competition results in little paternity bias (i.e., a “fair raffle” due to sperm mixing and/or female control; Parker, 1990; Simmons, 2001). As this would result in each male siring a portion of a female's brood, it may allow sufficient paternity confidence to facilitate the evolution of male nutritional investments in offspring (Sakaluk, 1986). In addition to this, selection for sperm competition mechanisms that result in strong paternity bias such as sperm displacement or inducing female refractory periods (Simmons, 2001) is probably undermined in systems where females rely on gifts for survival or egg development as they tend to mate frequently to obtain male‐supplied nutrition prior to oviposition. Under this hypothesis, we expect there to be shared or near‐equal paternity rather than a strong bias in insect systems that invest in offspring via nuptial feeding.

Most sperm competition research with arthropods has focused on paternity outcomes when each female is mated to two males in the lab. However, hypotheses about relative paternity are ideally tested using matings from the wild. These studies, typically using microsatellite markers of paternity, are less common and have shown variation in fertilization patterns (Frentiu & Chenoweth, 2008; Good et al., 2006; Simmons, 2001, 2007). In studies of species with no mate feeding, analysis of offspring from wild‐caught females has revealed evidence of high paternity bias: in fruit flies (*Drosophila melanogaster*; Imhof et al., 1998 and *Drosophila serrata*; Frentiu & Chenoweth, 2008), the tobacco fly (*Bactrocera cacuminata*; Song et al., 2007), and several species of gryllid crickets (*Gryllus bimaculatus*; Bretman & Trezenga, 2005, *Telogryllus commodus*, *Telogryllus oceanicus*; Simmons & Beveridge, 2010, and *Laupala cerasina*; Turnell & Shaw, 2015). In comparison, in species with mate feeding, studies suggest there is little paternity bias and no last male sperm precedence in wild‐caught females, including ladybird beetles (*Adalia bipunctata*; Haddrill et al., 2008), a katydid (*Requena verticalis*; Simmons, 2007), and a fruit fly (*Drosophila mojavensis*; Good et al., 2006). However, for two other gift‐giving katydids, *Pholidoptera griseoaptera* (Parker et al., 2017) and *Ephippiger ephippiger* (Hockham et al., 2004), high paternity bias has been reported. The findings for these two species may be explained by the methods used to measure paternity bias. As in most other studies, Hockham et al. (2004) measured the paternity skew among successful sires ($\sum {\left(\text{proportion offspring sired}\right)}^{2}$; Starr, 1984) in *E. ephippiger*, but did not include an estimate of the number of failed inseminations (males siring no offspring), which is an important component of paternity bias (Bretman & Trezenga, 2005; Gwynne & Lorch, 2012). Although both metrics were used to assess paternity bias in *P. griseoaptera*, analyses were conducted only on eggs showing embryonic development (less than half a clutch) as undeveloped eggs require several winters (diapause triggers) to hatch (Hartley & Warne, 1972; Ingrisch, 1986).

**FIGURE 1:**
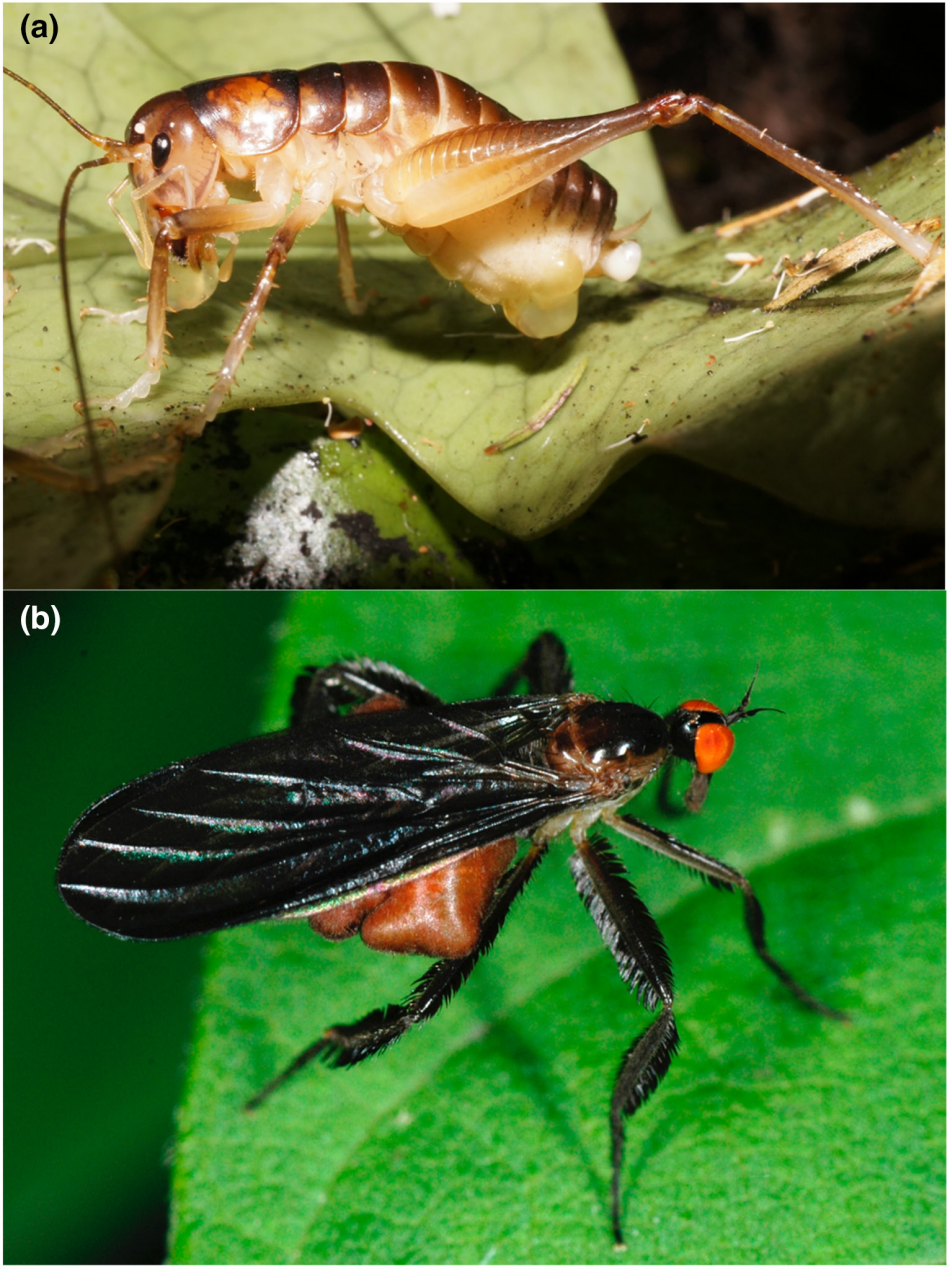
(a) Female ground weta (*Hemiandrus pallitarsis*) just after mating, eating part of the spermatophylax gift. One lobe remains adhered to her abdomen, and the globular sperm package is visible at the end of her abdomen. Photo by Darryl Gwynne. (b) Female dance fly (*Rhamphomyia longicauda*) showing inflated abdomen and leg‐scale ornaments. Photo by Heather Proctor.

In the current study, we investigate the outcome of sperm competition using wild‐caught insects of two species from different orders where males provide ornamented females with valuable nuptial gifts: the long‐tailed dance fly, *Rhamphomyia longicauda* (Diptera: Empidae) and an orthopteran ground weta, *Hemiandrus pallitarsis* (Orthoptera: Anostostomatidae) (Figure 1), in which we analyze relative paternity for all of a female's offspring (her lifetime reproduction). In both these species, nuptial gifts represent a paternal investment because females rely on them for successful offspring production (Browne, 2021; Downes, 1970; Gwynne, 2004; Hunter & Bussière, 2019) and appear to compete for matings, in part indicated by secondary sexual traits (Funk & Tallamy, 2000; Gwynne, 2004, 2005). If low paternity bias (i.e., fair raffle; Parker, 1970) helps facilitate the evolution of paternal investments by increasing confidence of paternity (i.e., reducing the chance of cuckoldry), we predict that there will be reduced paternity bias in both ground weta and dance flies, especially when compared with insect species that do not provide nuptial gifts. Using several common metrics to measure bias, we expect to observe (1) few males excluded from fathering offspring, (2) similar paternity shares among sires, and (3) little or no evidence of last‐male sperm precedence.

## 2 MATERIALS AND METHODS

### 2.1 Study species

Males of New Zealand “short‐tailed” ground weta provide females with spermatophylax meals consisting of gelatinous seminal secretions that are adhered to her mid‐abdomen, separate from the sperm‐containing ampulla (Gwynne, 2002, 2004, 2005). Female *H. pallitarsis* mate multiply to obtain these gifts (Browne, 2021), probably to help them survive a period of 5–6 months without food when they care for eggs laid in an underground brood chamber (hence their “short‐tail” reduced ovipositor) before dying (Gwynne, 2004). Females that obtain more gifts (mates) produce a greater number of surviving offspring (Browne, 2021) and apparently compete for mates as they possess an “ornamental” secondary genitalic device (accessory organ: Gwynne, 2004, 2005). This device is inserted between two main parts of the male genitalia as the gift is deposited (Gwynne unpublished) and appears to be under sexual selection (Browne, 2021). Because ground weta females lay all their eggs following a month‐long mating season (Gwynne, 2004), there is high potential for sperm competition, and we are able to measure paternity from a female's lifetime production of offspring.

Male empid dance flies, *Rhamphomyia longicauda*, catch prey (adults of small aquatic insects), which they transfer to females in exchange for mating within swarms (Funk & Tallamy, 2000). As females do not hunt on their own, they rely on these mating gifts to obtain protein for egg development (Downes, 1970; Hunter & Bussière, 2019) and mate multiply within a mating season (Downes, 1970; Herridge, 2016). Swarming females possess two sex‐specific ornaments (pinnate scales on the legs and inflated abdominal sacs) that function in attracting prey‐carrying males that are available only during swarming (Cumming, 1994; Funk & Tallamy, 2000); about an hour each dusk and dawn. Although studies have not shown directional sexual selection on females in this species (See Herridge, 2016; Wheeler et al., 2012), there is evidence that males prefer females with larger ornaments (Funk & Tallamy, 2000; Murray et al., 2018), even though ornament size correlates weakly with egg number and size (Funk & Tallamy, 2000; Wheeler, 2008). *R. longicauda* is well suited for testing our hypothesis, since females possess a single sclerotized sperm storage organ, in contrast to the multi‐channeled structure of many other dipterans (Pitnick et al., 1999; Puniamoorthy et al., 2010), creating high potential for sperm displacement and thus biased paternity in favor of the last male (Simmons, 2001).

### 2.2 Collection and rearing

At the end of the mating season, we collected 10 *H. pallitarsis* pairs (females and their last mate) in 2002 and another 17 in 2017 from Kiriwhakapapa trail near Masterton, NZ (−40.807627, 175.546532) and two private gardens in Palmerston North, NZ (−40.413909, 175.662814). Males were preserved at −20°C for DNA analysis, and live females were transported to our lab in Canada to oviposit eggs. This was an extended process that involved placing females in artificial brood chambers formed in potter's clay (2002) or soil (2017) and allowing 2–3 months for females to lay eggs, as well as an additional 5–6 months for eggs to mature. While in the brood chambers, we exposed females to typical winter temperatures, which were based on New Zealand weather records. All but three females laid eggs; however, these only developed in 19 of the broods, apparently due to female mortality. After eggs began hatching, we froze (at −20°C) the mothers and offspring (hatched nymphs or eggs with eye spots visible through the chorion) from 17 broods, excluding two broods where less than five eggs developed. We then extracted the sperm storage organ from all females, isolated the contents using >70% ethanol, which causes sperm to harden into a pellet (Tripet et al., 2001), and stored them at −20°C.

Similarly, we collected *R. longicauda* mating pairs (in copula, *n* = 131) from mating swarms near the Credit River, Glen Williams, Ontario, Canada (43.6865660, −79.9260960) from mid‐June to mid‐July of 2017. Males were killed and preserved via freezing while females were placed in individual plastic containers and kept at ambient temperatures (20–25°C) to lay eggs. We ensured the containers stayed moist by spraying them with water daily and provided females with fruit fly media, leaves, and moist cotton as oviposition location in the wild is unknown. Eighty‐eight females died without ovipositing, but 43 females laid 1–2 batches of eggs (1–89 eggs overall) on the sides of the containers before dying, typically within 3–4 days of capture. Dance fly eggs, which do not require maternal care for survival, were collected and stored in Petri dishes lined with damp filter paper. They were left to develop at room temperature for up to 6 days, at which point those showing signs of maturation (i.e., darkening of the chorion, visible mouthparts, or hatching) were frozen at −20°C for genetic analysis. We removed female's sperm storage organ and again used strong ethanol (>70%) to isolate the sperm pellet from female tissue (as in Tripet et al., 2001).

### 2.3 DNA extraction and microsatellite analysis

For *H. pallitarsis*, DNA was extracted from offspring (whole nymph bodies or developed eggs; 489 from 17 broods) and the hind leg or head of adults (17 females and 17 males) using a Proteinase K–based extraction method with multiple ethanol washes. DNA was extracted from sperm pellets (*n* = 17) using a Qiagen DNeasy Blood and Tissue Extraction kit with the addition of 12 μl DTT to each sample to improve lysis of sperm cells. Unfortunately, DNA extractions proved to be much more difficult in dance flies. While we were able to utilize the same Proteinase K–based method to extract DNA from the head and thorax of *R. longicauda* adults (11 females and 11 males), we had difficulty extracting sufficient quantities of DNA from offspring (whole developed eggs or first instar larvae). We eventually had some success using a Chelex‐based extraction with an initial homogenization step (359 offspring from 11 broods); however, we were unable to extract DNA from sperm pellets in quantities sufficient for PCR, despite using a QIAamp DNA Mirco kit with the addition of DTT as in Herridge (2016).

In both ground weta and dance flies, we genotyped samples at four loci using fluorescent labeled microsatellite markers. Markers for *H. pallitarsis* were developed by Genetic Marker Services, Brighton, UK (Table 1), and characterized in two populations (see Browne, 2021). We conducted PCR in two multiplex reactions, each containing 5.75 μl sterile filtered water, 1 μl 10X PCR reaction buffer, 0.2 μl 10 mM dNTP mixture, 0.5 μl of forward primer (with fluorescent labels HEX or 6‐FAM), 0.5 μl reverse primer, and 1 μl of template DNA. Thermocycling was done using an Eppendorf Mastercycler with the following temperature regime: 2 min at 94°C followed by 35 cycles of 30 s at 94°C, 30 s at 60°C, and 15 s at 72°C, then an additional 3 min at 72°C. Markers for *R. longicauda* were developed and characterized by E. J. Herridge and L. Bussière at University of Stirling for use with the same study population (Table 2; Herridge, 2016). We again conducted PCR in two multiplex reactions, this time each containing 5 μl sterile filtered water, 1 μl 10X PCR reaction buffer, 0.1 μl 50 M MgCl_2_, 0.2 μl 10 mM dNTP mixture, 0.75 μl forward primer (with fluorescent labels HEX or 6‐FAM), 0.75 μl reverse primer, and 2 μl of template DNA (1 μl for adults). The thermocycling regime was adapted from Herridge (2016) and included 5 min at 95°C followed by 40 cycles of 94°C for 30 s, 56°C for 30 s, 72°C for 25 s, then an additional 72°C for 10 min. We sent all PCR products to The Centre for Applied Genomics in Toronto, Ontario, for fragment analysis on an Applied Biosystems 3730xl capillary sequencer. The resulting electropherograms were examined using GeneMarker V1.97, which allowed us to identify fluorescence peaks, representing alleles.

**TABLE 1:** Microsatellite markers used to genotype *Hemiandrus pallitarsis*

Locus	Repeat motif	Floro‐label	Multiplex
wet80	(AG)14	HEX	1
wet81	(GA)16	HEX	2
wet89	(TC)41	6‐FAM	1
wet83b	(AC)6	6‐FAM	2

*Note*: See Browne (2021) for details.

**TABLE 2:** Microsatellite markers used to genotype *Rhamphomyia longicauda*

Locus	Repeat motif	Floro‐label	Multiplex
RL1AXKU5	(TA)9	6‐FAM	1
RL1BHDMD	(AT)9	HEX	1
RL1BWMXW	(TA)11	HEX	2
RL2F2Z0L	(AT)9	6‐FAM	2

*Note*: See Herridge (2016) for details.

### 2.4 Paternity analysis

Because of difficulties genotyping dance fly offspring, methods of paternity analysis differed between the two species. In ground weta, we used data from 11 to 51 (mean: 28.8) offspring (95% of those collected) to estimate the number of sires in each brood (*n* = 17). We first estimated the minimum sire number using GERUD 2.0 (Jones, 2005), a parentage program that computes the minimum father combination given the offspring genotypes across multiple loci. Offspring that shared an allele with a female's last mate at all loci were considered to be fathered by this male. Additionally, we estimated the most likely number of sires in each brood using COLONY Version 2.0.6.6 (Jones & Wang, 2010). This program uses population allele frequencies (unpublished data) to determine the most likely parental configuration. A female's last mate was included in COLONY as a candidate father to determine which offspring were most likely sired by him (See Turnell & Shaw, 2015 for comparison of parentage programs). We then estimated the number of males that failed to sire offspring by identifying alleles in a female's sperm storage organ that were not present in any of the offspring. We used allele counting (assumes heterozygosity; number of unique paternal alleles present in offspring divided by two) to estimate the minimum number of additional males for each brood.

In the dance flies, our analysis was more limited. While we genotyped 11–58 (mean: 32.6) offspring from each brood (*n* = 11), this only accounted for a portion (45% on average) of those produced, and in some cases only included data for two loci. Further, we could not determine which male alleles remained in the sperm storage organ, as we were unable to extract DNA from female‐stored sperm pellets. Using the available offspring data for 11 dance fly broods, we first counted the unique paternal alleles present in offspring and divided by 2 to estimate the minimum number of sires at each locus. Next, we determined the most likely sire configuration using COLONY (Jones & Wang, 2010), again including the female's last mate as a candidate father. We were unable to incorporate population allele frequencies into estimates of sire number (as in weta) because they did not include all alleles present in offspring (likely a consequence of high microsatellite polymorphism). In the absence of DNA from female sperm stores, we compared our estimates of sire number with estimates of mate number for this population (determined by Herridge, 2016) to assess whether males likely experience paternity loss. We used the estimate of minimum sire number (allele counting from offspring) so that this would be comparable with Herridge's (2016) estimate of minimum mate number (allele counting from the sperm storage organ).

### 2.5 Statistical analysis

In both ground weta and dance flies, we tested whether paternity patterns deviated from a “fair raffle” scenario (Parker, 1990), in which males sire equal (or near equal; Herridge, 2016) proportions of offspring. Among the multiply mated females, we measured the paternity skew using Starr's (1984) measure ($\sum {\left(\text{proportion offspring sired}\right)}^{2}$, in which a value of 1 represents complete paternity bias in favor of a particular male), and plotted this against sire number. For both weta and dance flies, we determined whether the observed paternity skew was significantly higher than the skew expected if offspring were distributed evenly to all sires using the 95% confidence intervals of the best fit line (as in Simmons & Beveridge, 2010; Turnell & Shaw, 2015) as well as a paired *t*‐test. Finally, we tested for evidence of last‐male sperm precedence in both *H. pallitarsis* and *R. longicauda* by determining whether a female's last mate fathered a greater proportion of offspring than other males, or than would be expected by equal shares. One weta brood was removed from this analysis because the last male did not appear to successfully inseminate the female, as his alleles were not found in either her offspring or stored sperm.

## 3 RESULTS

### 3.1 Sire number

Among the 17 *H. pallitarsis* broods, the minimum sire number (GERUD) averaged 3 ± 1.6 SD (range 1–6). Estimates of most likely sire number (COLONY) were much higher with an average of 5.9 ± 2.4 SD (range 2–10). Regardless of the method used, the number of sires detected did not change with the number of offspring tested (GERUD: *ß* = .03, *R* ^2^ = .03, *p* = .525, COLONY: *ß* = .08, *R* ^2^ = .12, *p* = .173). Most females mated multiply and had offspring sired by at least two males (GERUD: 83%, COLONY: 100%).

We estimated sire number in dance flies using the 11 broods from which we were able to genotype offspring. When we used the conservative method of allele counting, the number of sires averaged 3.6 ± 1.3 SD and ranged from 2 to 6 sires. Estimates of sire number were much higher when using COLONY, which averaged 13.5 ± 5.9 SD sires and ranged from 7 to 26. In dance flies, the number of offspring tested did influence the number of sires we were able to detect. We found a significant positive relationship between the number of offspring genotyped and number of sires detected for both of our estimates; however, this relationship was much stronger when using COLONY rather than allele counting (Allele counting: *ß* = .07, *R* ^2^ = .69, *p* = .002; COLONY: *ß* = .34, *R* ^2^ = .87, *p* = < .0001). Regardless of the method used to estimate sire number, all females mated multiply and had offspring fertilized by at least two sires.

### 3.2 Paternity bias

**FIGURE 2:**
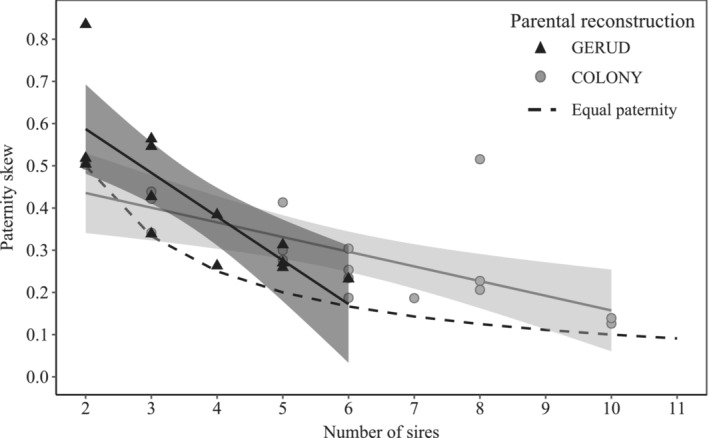
Paternity skew occurring in broods of multiply mated *Hemiandrus pallitarsis* females, plotted against the minimum number of sires (parental reconstruction determined using GERUD; *n* = 13) or most likely number of sires (COLONY; *n* = 17) estimated from microsatellite analysis of offspring. The regression lines (solid) show the relationship between number of sires and observed paternity skew with 95% confidence intervals showing deviation from the null skew expected when all sires father an equal number of offspring (dashed line).

Additional alleles, suggestive of males that mated but did not sire offspring, were found in the sperm storage organ of five female ground weta; however, these rarely represented more than one male per brood. On average, 85.4% (binomial 95% CI: 74.6%, 93.1%) of the males that inseminated a female fathered offspring. The rate of paternity success was similar (mean: 88.6%, binomial 95% CI: 81.9%, 93.5%) when using COLONY to estimate sire number. Despite this, paternity shares among weta sires were still biased. Overall, the paternity skew (degree to which paternity is unevenly distributed) was significantly higher than would be expected if all sires fathered an equal number of offspring (Figure 2; one‐tailed paired *t*‐test, mean difference = 0.10, *t* = 3.72, df = 12, *p* = .001). This result was consistent when using COLONY to estimate the most likely sire number (Figure 2; one‐tailed paired *t*‐test, mean difference = 0.09, *t* = 4.17, df = 16, *p* = .0004). Paternity skew declined linearly with the number of sires (GERUD: *ß* = −.10, *R* ^2^ = .61, *p* < .001; COLONY: *ß* = −.03, *R* ^2^ = .42, *p* = .003), although this was not a better fit than an inverse (non‐linear, *y* = 1/*x*) relationship (GERUD: *R* ^2^ = .64, *p* < .001, δAIC < 1.0; COLONY: *R* ^2^ = .43, *p* = .002, δAIC < 1.0).

**FIGURE 3:**
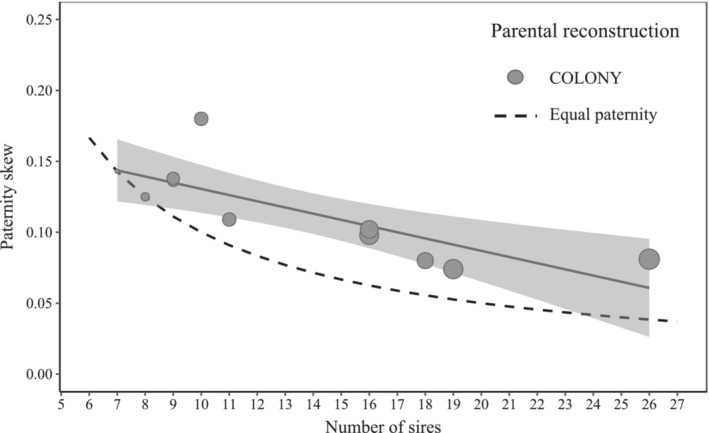
Paternity skew among offspring of female *Rhamphomyia longicauda* (*n* = 11) plotted against the most likely number of sires (determined from microsatellite analysis of offspring; COLONY). The regression line (solid) shows the linear relationship between number of sires and observed paternity skew with 95% confidence intervals showing deviation from the null skew expected when all sires father an equal number of offspring. Size of points represents the number of offspring analyzed for each estimate of paternity skew, which has a positive effect on the number of sires detected (see main text).

For dance flies, although we could not genotype the contents of a female's sperm storage organ to estimate the proportion of failed matings, we found paternity skew among sires, similar to the weta. Using the most likely paternal configuration (COLONY), the paternity skew was determined to be significantly higher than would be expected if all sires fathered an equal number of offspring (Figure 3; one‐tailed paired *t*‐test, mean difference = 0.03, *t* = 6.09, df = 10, *p* = <.0001). Again, paternity skew declined linearly with number of sires (*ß* = −.004, *R* ^2^ = .59, *p* < .003), but this was no better fit than an inverse (nonlinear, *y* = 1/*x*) relationship (*R* ^2^ = .57, *p* = .004, δAIC < 2.0).

### 3.3 Last‐male sperm precedence

**FIGURE 4:**
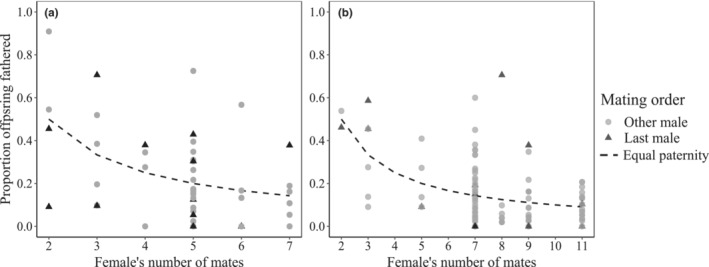
Proportion of offspring fathered by last male relative to all other competing males in *Hemiandrus pallitarsis* ground weta using estimates from (a) GERUD (minimum sire configuration; *n* = 12) and (b) COLONY (most likely sire configuration; *n* = 16). Data are shown relative to the proportion of offspring each male would sire if paternity shares were equal (null paternity).

In ground weta, a female's last mate did not consistently have a fertilization advantage. Based on the minimum father combination (GERUD), last males were seldom the most successful male (42% of broods) but fathered offspring in all but two broods (Figure 4a). Overall, a female's last mate did not father a significantly greater proportion of offspring than previous males (Welch two sample *t*‐test: *t* = 0.37, df = 16.89, *p* = .357) or that expected under a fair raffle scenario (equal shares among mates) (one‐tailed paired *t*‐test: *t* = −0.70, df = 11, *p* = .751). When using COLONY to reconstruct the mostly likely paternal configuration, the last male was excluded from a further five broods (fathering the majority of offspring in only 25% of broods; Figure 4b) and overall did not father a significantly greater proportion of offspring than previous mates (Welch two sample *t*‐test: *t* = 0.70, df = 16.52, *p* = .247) or that expected under a fair raffle scenario (equal shares among mates) (one‐tailed paired *t*‐test: *t* = −0.03, df = 15, *p* = .513).

**FIGURE 5:**
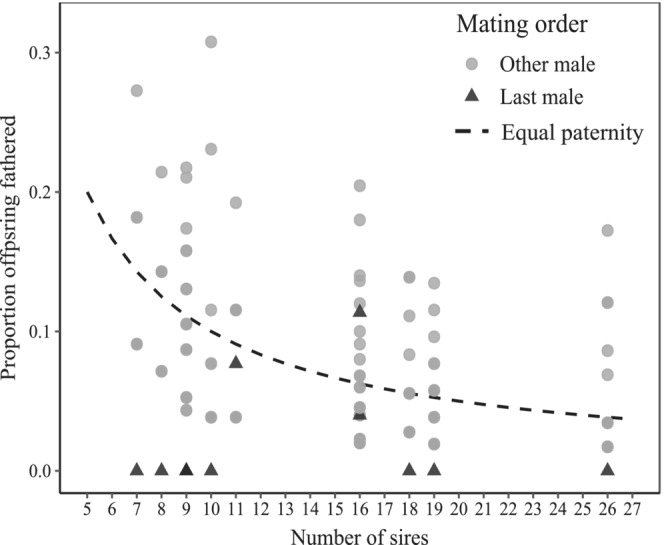
Proportion of offspring fathered by last male relative to other siring males in *Rhamphomyia longicauda* dance flies (using COLONY to estimate the most likely sire configuration; *n* = 11). Data are shown relative to the proportion of offspring each male would sire if paternity shares were equal (the null).

In the dance flies, there was no evidence of last‐male sperm precedence in our samples. Although last‐male alleles were detected in offspring from each brood, the most likely paternal configuration (COLONY) suggested that 73% of these males did not fertilize any offspring and none sired the majority (Figure 5). Overall, last males fathered a significantly lower proportion of offspring than previously mated males (Welch two sample *t*‐test: *t* = −4.07, df = 13.58, *p* = .001) and that expected under a fair raffle scenario (equal shares among sires) (one‐tailed paired *t*‐test: *t* = −3.74, df = 10, *p* = .004).

## 4 DISCUSSION

For two insect species where sexually selected, ornamented females rely on nutrition provided by males during mating, we show evidence of multiple (shared) paternity with few males excluded from fathering offspring and no last‐male advantage. While we did observe skewed paternity (unequal paternity shares) among sires, paternity bias appears to be overall lower compared with insects that do not contribute paternal effort. While each male will only sire a portion of a female's brood, we suggest that low paternity bias reduces the chance of complete paternity loss (thus increased confidence in paternity), which may facilitate the evolution of systems where all mating males make paternal investments via nuptial gifts (Sakaluk, 1986). While this is contrary to some predictions about increased paternity bias through last‐male sperm precedence in species that invest in nuptial gifts (Gwynne, 1984a; Simmons, 2001), it is comparable with patterns found in many paternal care systems where females distribute their eggs between several males, each of which has high paternity confidence (Berglund et al., 1988).

In ground weta (*H. pallitarsis*), paternity estimates using a single brood of eggs (a female's lifetime offspring production) revealed a level of paternity confidence in which most investing males sired offspring. Although paternity was significantly skewed among sires, we found multiple paternity in most broods, with an average of 3.0 ± 1.6 SD sires (most likely estimate: 5.9 ± 2.4 SD), and no evidence of last‐male sperm precedence. In dance flies (*R. longicauda*), multiple paternity was observed in all clutches, with offspring being shared between a high number of sires (minimum mean: 3.6, most likely mean: 13.5). Paternity was again significantly skewed among sires, but we found no evidence that this was influenced by last‐male sperm precedence. Paternity estimates were constrained in dance flies due to incomplete genotypic data (Gerlach et al., 2012; Jones & Wang, 2010) and an inability to incorporate population allele frequencies into the paternal configuration (COLONY). This may help explain the positive relationship between the number of offspring analyzed and sires detected, as well as the high the upper limit on female mating rate (26 sires). This rate of polyandry is not unexpected, however, given the biology of dance flies. Individually marked female *R. longicauda*, which may live several weeks (unpublished data), have been observed returning to (dawn and dusk) mating swarms, up to two times per day (R. Murray, personal communication). Although we could not measure the proportion of males that failed to sire offspring in dance flies, this was assessed indirectly by determining if the number of mates (estimated in Herridge, 2016 using the same study population) exceeded the number of sires. While the number of unique paternal alleles in each clutch showed females had an average of 3.6 ± 1.3 SD sires (range 2–6), Herridge's (2016) analysis of alleles present in stored sperm indicated that *R. longicauda* females mated with a mean of 2.5 ± 0.15 SD (range 1–6) males. Based on these data, the number of mates does not exceed the number of sires, suggesting that few or no males experience paternity failure in *R. longicauda*.

Similar to our results, all other reports of paternity in insects with valuable nuptial gifts, including katydids (*P. griseoaptera*; Parker et al., 2017, *E. ephippiger*; Hockham et al., 2004, and *R. verticalis*; Simmons, 2007) a beetle (*A. bipunctata*; Haddrill et al., 2008), and a fly (*D. mojavensis*; Good et al., 2006), found some evidence of paternity skew among successful sires. Skewed paternity has also been found in species that lack nuptial gifts and have no apparent sexual competition among females, including flies (*D. melanogaster*; Imhof et al., 1998, *D. serrata*; Frentiu & Chenoweth, 2008, and *B. cacuminata*; Song et al., 2007), and crickets (*G. bimaculatus*; Bretman & Trezenga, 2005, *T. commodus*, *T. oceanicus*; Simmons & Beveridge, 2010, and *L. cerasina*; Turnell & Shaw, 2015); However, one main difference appears to be the higher proportion of mating males that do not sire any offspring (paternity failure) in no‐gift species relative to gift species. For example, analysis of stored sperm in gryllid crickets revealed that only 60% of a female's mates sired offspring in *T. commodus*, 75% in *T. oceanicus* (Simmons & Beveridge, 2010), 51%–66% in *L. cerasina* (Turnell & Shaw, 2015), and approximately 45%–85% in *G. bimaculatus* (when comparing sires to mating rates; Bretman & Trezenga, 2005). The probability of siring offspring was greater in at least two species (not measured in *D. mojavensis*; Good et al., 2006) in which females eat all (Perry & Tse, 2013) or a specialized part (spermatophylax: Gwynne, 1984b) of nutritious spermatophores. In the katydid *R. verticalis*, all males that inseminated a female sired offspring (Simmons, 2007), and in *A. bipunctata*, the number of mates did not exceed the number of sires (Haddrill et al., 2008), suggesting a low rate of paternity failure. This is similar to our findings in the ground weta, where 85%–89% of mates sire offspring, as well as the dance flies, where the number of mates (Herridge, 2016) did not exceed the number of sires.

In most insect species, some level of paternity failure may be expected due to the occurrence of infertile males or those that produce non‐viable eggs (Garcia‐González, 2004; Simmons & Beveridge, 2010). While these cases probably inflate the number of males that appear to be unsuccessful in sperm competition (Garcia‐González, 2004, 2008), this measure is an important indicator of paternity bias (Bretman & Trezenga, 2005). In particular, cases of zero fitness are especially important in driving variation in reproductive success (sexual selection) on males (see Gwynne & Lorch, 2012; Shuster & Wade, 2003). Unfortunately, the proportion of mates that sire offspring is not commonly reported in studies of paternity, which may explain why high bias (interpreted from paternity skew alone) was reported for *E. ephippiger* (Hockham et al., 2004), a katydid with extremely large spermatophylax gifts (Vahed & Gilbert, 1996). This was not the case in another gift‐giving katydid species, *P. griseoaptera* (Parker et al., 2017), however, as analysis of stored sperm indicated high rates of paternity failure in addition to unequal paternity among sires (skew). Notably, one reason for this finding may be the low proportion of offspring sampled, potentially underestimating the percentage of successful sires. As *Pholidioptera* (and also *Ephippiger*) require several winters for eggs laid late in the season to hatch (Hartley & Warne, 1972; Ingrisch, 1986), paternity analyses were conducted on a relatively small portion (20 per brood) of the offspring that reached the whole‐embryo stage in the lab (about 40% of viable eggs). The remaining eggs, in addition to those requiring additional winters to continue development, were not included (Parker et al., 2017), potentially inflating the number of paternity‐losing males (Fritzsche & Arnqvist, 2013; Gerlach et al., 2012; Imhof et al., 1998). Alternatively, high paternity bias may be less costly in this species if the male's spermatophylax gift primarily functions in maximizing sperm transfer, by extending the duration of spermatophore attachment (mating effort) rather than investment in a particular female and her offspring (Will & Sakaluk, 1994; reviewed in Vahed, 1998). Indeed, the size of the spermatophylax gift is small in *P. griseoaptera* (7% of his body weight) relative to some other katydids (Vahed & Gilbert, 1996), and male refractory periods are shorter than those of females (Parker et al., 2017).

Despite the low rate of paternity failure, there was significant paternity skew among sires in our species, which is consistent with findings in other insects regardless of whether they donate nuptial gifts. The observed paternity skew in our study did not appear to be related to last‐male sperm precedence (common in insects; Simmons, 2001). We note, however, that in the dance flies, our collection methods (mating pairs in flight) may have reduced copulation duration and thus the degree of sperm transfer from a female's last mate. This unlikely to be an issue in the ground weta as mated pairs were collected at the end of copulation when the female had nearly finished consuming the spermatophylax gift. The large, elastic sac of the sperm storage organ of ensiferan Orthoptera such as ground weta likely facilitates sperm mixing, even with frequent mating (Simmons, 2001). In contrast, dance flies have a sclerotized, non‐flexible sperm storage organ that would be expected to cause sperm displacement, with sperm from previous males being indirectly flushed from the storage organ (Simmons, 2001). Indeed, Herridge (2016) found that stored sperm tended to be dominated by a particular male in *R. longicauda*, but this could not be connected to mating order or the resulting paternity shares. Given the lack of last‐male sperm precedence, there are several possibilities that may explain the occurrence of paternity skew in our species, despite the prediction of reduced bias. First, random error during fertilization (e.g., slow or “sloppy” sperm mixing or sperm loss; Simmons, 2001) is likely to cause small differences in paternity success among males that results in deviation from a perfect fair raffle (Herridge, 2016). Thus, while the observed skew was significant in both the ground weta and dance fly, this may not represent much more bias than would be expected at under a “noisy fair raffle” scenario (Herridge, 2016). Alternatively, the observed skew could be caused by differences in male phenotype related to variation in sperm viability (Garcia‐González, 2004, 2008) or the number of sperm transferred (Parker et al., 2017; Simmons, 2001). In particular, gift size or quality may be expected to play a role in fertilization success by increasing copulation duration (reviewed in Vahed & Gilbert, 1996; Haddrill et al., 2008; Vahed, 1998) or influencing patterns of sperm storage by the female (Albo et al., 2013; Engels & Sauer, 2006; Fedina, 2007). Because the spermatophylax is secreted by the male's accessory glands, variation in ground weta gift quality is expected to be mediated by differences in physiological condition (reviewed in Lewis et al., 2014). Despite this, preliminary evidence suggests that male size (indicator of condition; Emlen, 1997; Emlen et al., 2012; Johnstone et al., 2009) does not influence the number of offspring sired in *H. pallitarsis* (unpublished data). Differences in copulation duration may be expected in ground weta, however, since sperm transfer ends in ensiferan Orthoptera when the externally placed sperm ampulla (Brown & Gwynne, 1997) is removed. As male short‐tailed ground weta guard the female while she consumes the spermatophylax gift, this may reduce the incidence of premature removal by their mates after copulation (Gwynne, 2004). On the other hand, mating gifts in dance flies consist of prey items (usually small insects) captured by the male (Cumming, 1994; Downes, 1970). As males provide females with a diversity of different prey‐types and sometimes even consume parts of the gift before entering mating swarms (as in *Rhamphomyia sulcata*; LeBas et al., 2004), dance fly gifts have the potential to vary considerably in protein content or handling times for the female. This may allow greater variation in gift quality and male effort relative to weta and may be expected to influence paternity by affecting the duration of female feeding and thus the amount of sperm transferred.

While paternity sharing in our two study species reduces the maximum number of offspring that can be sired by individual males, it appears to assure some paternity, as the chance of siring no offspring is low. Thus, reduced paternity bias may be an important factor in the evolution of nutritious nuptial gifts (Sakaluk, 1986). Low levels of paternity bias may be expected in other species where females rely on costly mating gifts to produce offspring such as many katydids (Gwynne, 1981, 1984c, 1985, 1988; Simmons & Bailey, 1990) and other species of empidine dance flies (Diptera: Empididae; Bussière et al., 2008; Cumming, 1994; Downes, 1970; Murray et al., 2018; Wheeler et al., 2012). To help understand this relationship, future studies should focus on obtaining thorough and consistent measures of paternity bias using wild‐caught females (including estimates of failed inseminations) across a range of species.

## AUTHOR CONTRIBUTIONS

**Jessica H. Browne:** Conceptualization (equal); data curation (lead); formal analysis (lead); investigation (lead); methodology (lead); software (lead); writing – original draft (lead); writing – review and editing (equal). **Darryl T. Gwynne:** Conceptualization (equal); formal analysis (supporting); funding acquisition (lead); methodology (supporting); resources (lead); supervision (lead); writing – original draft (supporting); writing – review and editing (equal).

## Data Availability

The data that support the findings of this study are openly available in Dryad at https://doi.org/10.5061/dryad.nk98sf7wq.
